# Systematic Error Modeling and Bias Estimation

**DOI:** 10.3390/s16050729

**Published:** 2016-05-19

**Authors:** Feihu Zhang, Alois Knoll

**Affiliations:** Robotics and Embedded Systems, Technische Universität München, 80333 München, Germany; knoll@in.tum.de

**Keywords:** systematic error, bias, least square method

## Abstract

This paper analyzes the statistic properties of the systematic error in terms of range and bearing during the transformation process. Furthermore, we rely on a weighted nonlinear least square method to calculate the biases based on the proposed models. The results show the high performance of the proposed approach for error modeling and bias estimation.

## 1. Introduction

Interest in Cooperative Localization (C.L.) has grown exponentially in the past few years [1,2]. The concept of C.L. is demonstrated by collecting a large amount of data from heterogeneous sensors to improve the volume of surveillance and increase the estimation reliability [3]. This requires local sensors transforming their data to a common reference system for further processing. Due to the nonlinear issues, the transformations often introduce systematic errors [4,5]. Hence, the sensor registration algorithms are quite important.

Sensor registration mainly refers to the systematic errors, in contrast to the random errors. Various methods have been proposed including the centralized [6,7,8] and decentralized solutions [9,10,11,12]. In the centralized solution, the exact maximum likelihood method is proposed where the sensor measurements were first projected onto the local coordinate, and then transformed to the public region [13]. However, the errors introduced from stereographic projection suffer in both the local sensor and the public region. Regarding the decentralized solution, Lin *et al*. presented the bias estimation based on the local tracks at different frames [14]. This work considered both offset biases and scale biases and demonstrated some preliminary results. However, this technique estimates the biases during the filtering phases, which suffer from target maneuvers. Zhang *et al*. presented the bias estimation based on the probability hypothesis density filter, in which only the translational biases were considered [15].

In this paper, the statistic properties of the systematic errors are first analyzed, with respect to the mean and covariance. Then the weighted nonlinear least square method is utilized to estimate the corresponding biases in terms of range and bearing. The proposed solution also considers the maneuvers by normalizing the weights of the pseudo-measurements based on the covariances.

This paper is organized as follows: Section 2 investigates the statistic proprieties of the systematic errors. Section 3 introduces the nonlinear least square method to estimate the biases. Section 4 presents simulation results in synchronous sensors. Finally, the paper is concluded in Section 5.

## 2. Analysis of Systematic Error Properties

In this section, the systematic error is analyzed with respect to the expectation and covariance in terms of range and bearing.

### 2.1. Problem Statement

In cooperative localization, measurements are collected in a polar coordinate system which also contains both biases and random noises. The measured range $r_{m}$ and bearing $\theta_{m}$ are thus defined as

(1) $$r_{m}=\bar{r}+r_{b}+\tilde{r}$$

(2) $$\theta_{m}=\bar{\theta }+\theta_{b}+\tilde{\theta }$$

where $\bar{r}$ and $\bar{\theta }$ denote the ground truth, $r_{b}$ and $\theta_{b}$ denote the corresponding biases. $\tilde{r}$ and $\tilde{\theta }$ are assumed to be the Gaussian noises with zero mean and standard deviations $\sigma_{r}$ and $\sigma_{\theta }$. For the localization task, measurements from polar coordinates are transformed to Cartesian coordinates by using

(3) $$x_{m}=r_{m}\cos\theta_{m}$$

(4) $$y_{m}=r_{m}\sin\theta_{m}$$

### 2.2. True Systematic Error

The transformed measurement can also be represented as the combination of the true value and the systematic error.

(5) $$x_{m}=r_{m}\cos\theta_{m}=\left(\bar{r}+r_{b}+\tilde{r}\right)\cos\left(\bar{\theta }+\theta_{b}+\tilde{\theta }\right)=\bar{x}+\tilde{x}$$

(6) $$y_{m}=r_{m}\sin\theta_{m}=\left(\bar{r}+r_{b}+\tilde{r}\right)\sin\left(\bar{\theta }+\theta_{b}+\tilde{\theta }\right)=\bar{y}+\tilde{y}$$

where $\bar{x}=\bar{r}\cos\bar{\theta }$ and $\bar{y}=\bar{r}\sin\bar{\theta }$ denote the true values and $\tilde{x}$ and $\tilde{y}$ denote the systematic errors.

Rearranging Equtions (5) and (6), we have the representation of the systematic errors. Here we only show the systematic in *x* direction for simplification reasons.

(7) $$\tilde{x}=r_{m}\cos\theta_{m}-\bar{r}\cos\bar{\theta }=\bar{r}\left[A-\cos\bar{\theta }\right]+r_{b}\left[A\right]+\tilde{r}\left[A\right]$$

and

(8) $${\tilde{x}}^{2}={\bar{r}}^{2}{\left[A-\cos\bar{\theta }\right]}^{2}+r_{b}^{2}{\left[A\right]}^{2}+{\tilde{r}}^{2}{\left[A\right]}^{2}+2\bar{r}r_{b}\left[A-\cos\bar{\theta }\right]\left[A\right]+2\bar{r}\tilde{r}\left[A-\cos\bar{\theta }\right]\left[A\right]+2\tilde{r}r_{b}{\left[A\right]}^{2}$$

where

(9) $$A=\cos\bar{\theta }\cos\theta_{b}\cos\tilde{\theta }-\sin\bar{\theta }\sin\theta_{b}\cos\tilde{\theta }-\sin\bar{\theta }\cos\theta_{b}\sin\tilde{\theta }-\cos\bar{\theta }\sin\theta_{b}\sin\tilde{\theta }$$

Instead of calculating systematic error by using ground-truths, biases and noises, the statistics properties are considered where the random noises are eliminated.

The expectation and covariance of the systematic error can be explicitly calculated with following equations:

(10) $$E\left[\cos\tilde{\theta }\right]=e^{-\frac{\sigma_{\theta }^{2}}{2}}$$

(11) $$E[\sin\tilde{\theta }]=0$$

(12) $$E\left[\cos^{2}\tilde{\theta }\right]=\frac{1+e^{-2\sigma_{\theta }^{2}}}{2}$$

(13) $$E\left[\sin^{2}\tilde{\theta }\right]=\frac{1-e^{-2\sigma_{\theta }^{2}}}{2}$$

(14) $$E[\sin\tilde{\theta }\cos\tilde{\theta }]=0$$

The statistic properties of the systematic error are summarized as

(15) $$E\left[\tilde{x}\right]=\bar{r}\left[\cos\bar{\theta }\cos\theta_{b}e^{-\sigma_{\theta }^{2}/2}-\sin\bar{\theta }\sin\theta_{b}e^{-\sigma_{\theta }^{2}/2}-\cos\bar{\theta }\right]+r_{b}\left[\cos\bar{\theta }\cos\theta_{b}e^{-\sigma_{\theta }^{2}/2}-\sin\bar{\theta }\sin\theta_{b}e^{-\sigma_{\theta }^{2}/2}\right]$$

and

(16) $$\begin{array}{c} \begin{array}{c} E\left[{\tilde{x}}^{2}\right]={\bar{r}}^{2}\left[B+\cos^{2}\bar{\theta }-2\cos^{2}\bar{\theta }\cos\theta_{b}e^{-\sigma_{\theta }^{2}/2}+\sin2\bar{\theta }\sin\theta_{b}e^{-\sigma_{\theta }^{2}/2}\right]+r_{b}^{2}B+\sigma_{r}^{2}B\\ +2\bar{r}r_{b}\left[B-\cos^{2}\bar{\theta }\cos\theta_{b}e^{-\sigma_{\theta }^{2}/2}+\sin2\bar{\theta }\sin\theta_{b}e^{-\sigma_{\theta }^{2}/2}/2\right] \end{array} \end{array}$$

where B equals

$$\begin{array}{c} B=\cos^{2}\bar{\theta }\cos^{2}\theta_{b}\left(1+e^{-2\sigma_{\theta }^{2}}\right)/2+\sin^{2}\bar{\theta }\sin^{2}\theta_{b}\left(1+e^{-2\sigma_{\theta }^{2}}\right)/2+\\ \sin^{2}\bar{\theta }\cos^{2}\theta_{b}\left(1-e^{-2\sigma_{\theta }^{2}}\right)/2+\cos^{2}\bar{\theta }\sin^{2}\theta_{b}\left(1-e^{-2\sigma_{\theta }^{2}}\right)/2-\sin2\bar{\theta }\sin2\theta_{b}e^{-2\sigma_{\theta }^{2}}/2 \end{array}$$

Similarly, the corresponding systematic error in *y* direction is calculated. Equation (15) and Equation (16) still depend on the ground-truths, which are unavailable in practice. Hence, the systematic errors are calculated again on condition of the measurements.

### 2.3. Systematic Error in Practice

Based on the Equation (15) and Equation (16), the conditional first and second order moments are calculated as

(17) $$\begin{array}{c} E\left[E\left[\tilde{x}\right]|r_{m},\theta_{m}\right]=r_{m}[\cos\left(\theta_{m}-\theta_{b}\right)\cos\theta_{b}e^{-\sigma_{\theta }^{2}}-\sin\left(\theta_{m}-\theta_{b}\right)\sin\theta_{b}e^{-\sigma_{\theta }^{2}}-\\ \cos\left(\theta_{m}-\theta_{b}\right)e^{-\sigma_{\theta }^{2}/2}]+r_{b}\cos\left(\theta_{m}-\theta_{b}\right)e^{-\sigma_{\theta }^{2}/2} \end{array}$$

(18) $$\begin{array}{c} E\left[E\left[{\tilde{x}}^{2}\right]|r_{m},\theta_{m}\right]=C\times \left[M+D-2D\cos\theta_{b}e^{-\sigma_{\theta }^{2}/2}+F\sin\theta_{b}e^{-\sigma_{\theta }^{2}/2}\right]+r_{b}^{2}\left[M\right]+\sigma_{r}^{2}\left[M\right]\\ +\left(2r_{b}r_{m}-2r_{b}^{2}\right)\left[M-D\cos\theta_{b}e^{-\sigma_{\theta }^{2}/2}+\frac{1}{2}F\sin\theta_{b}e^{-\sigma_{\theta }^{2}/2}\right] \end{array}$$

and

(19) $$\begin{array}{c} E\left[E\left[\tilde{y}\right]|r_{m},\theta_{m}\right]=r_{m}[\sin\left(\theta_{m}-\theta_{b}\right)\cos\theta_{b}e^{-\sigma_{\theta }^{2}}+\cos\left(\theta_{m}-\theta_{b}\right)\sin\theta_{b}e^{-\sigma_{\theta }^{2}}-\\ \sin\left(\theta_{m}-\theta_{b}\right)e^{-\sigma_{\theta }^{2}/2}]+r_{b}\sin\left(\theta_{m}-\theta_{b}\right)e^{-\sigma_{\theta }^{2}/2} \end{array}$$

(20) $$\begin{array}{c} E\left[E\left[{\tilde{y}}^{2}\right]|r_{m},\theta_{m}\right]=C\times \left[N+E-2E\cos\theta_{b}e^{-\sigma_{\theta }^{2}/2}-F\sin\theta_{b}e^{-\sigma_{\theta }^{2}/2}\right]+r_{b}^{2}\left[N\right]+\sigma_{r}^{2}\left[N\right]\\ +\left(2r_{b}r_{m}-2r_{b}^{2}\right)\left[N-E\cos\theta_{b}e^{-\sigma_{\theta }^{2}/2}-\frac{1}{2}F\sin\theta_{b}e^{-\sigma_{\theta }^{2}/2}\right] \end{array}$$

where

$$C=r_{m}^{2}+r_{b}^{2}+\sigma_{r}^{2}-2r_{m}r_{b}$$

$$D=\cos^{2}\left(\theta_{m}-\theta_{b}\right)\frac{1+e^{-2\sigma_{\theta }^{2}}}{2}+\sin^{2}\left(\theta_{m}-\theta_{b}\right)\frac{1-e^{-2\sigma_{\theta }^{2}}}{2}$$

$$E=\sin^{2}\left(\theta_{m}-\theta_{b}\right)\frac{1+e^{-2\sigma_{\theta }^{2}}}{2}+\cos^{2}\left(\theta_{m}-\theta_{b}\right)\frac{1-e^{-2\sigma_{\theta }^{2}}}{2}$$

$$F=\sin2\left(\theta_{m}-\theta_{b}\right)e^{-2\sigma_{\theta }^{2}}$$

$$M=D\cos^{2}\theta_{b}\frac{1+e^{-2\sigma_{\theta }^{2}}}{2}+E\sin^{2}\theta_{b}\frac{1+e^{-2\sigma_{\theta }^{2}}}{2}+E\cos^{2}\theta_{b}\frac{1-e^{-2\sigma_{\theta }^{2}}}{2}+D\sin^{2}\theta_{b}\frac{1-e^{-2\sigma_{\theta }^{2}}}{2}-F\sin2\theta_{b}\frac{e^{-2\sigma_{\theta }^{2}}}{2}$$

$$N=E\cos^{2}\theta_{b}\frac{1+e^{-2\sigma_{\theta }^{2}}}{2}+D\sin^{2}\theta_{b}\frac{1+e^{-2\sigma_{\theta }^{2}}}{2}+D\cos^{2}\theta_{b}\frac{1-e^{-2\sigma_{\theta }^{2}}}{2}+E\sin^{2}\theta_{b}\frac{1-e^{-2\sigma_{\theta }^{2}}}{2}+F\sin2\theta_{b}\frac{e^{-2\sigma_{\theta }^{2}}}{2}$$

Equations (17)–(20) successfully estimate the systematic error by using the original measurements. The uncertainty of the estimation is also calculated as

(21) $$var\left(\tilde{x}\right)=E\left[E\left[{\tilde{x}}^{2}\right]|r_{m},\theta_{m}\right]-E^{2}\left[E\left[\tilde{x}\right]|r_{m},\theta_{m}\right]$$

(22) $$var\left(\tilde{y}\right)=E\left[E\left[{\tilde{y}}^{2}\right]|r_{m},\theta_{m}\right]-E^{2}\left[E\left[\tilde{y}\right]|r_{m},\theta_{m}\right]$$

## 3. Bias Estimation Using Nonlinear Least Square Method

To calculate the biases, information from additional sensors are required. Suppose both the radar and GPS measurements (GPS only contains random noises) are provided, we have

(23) $$x_{m}^{r}-x_{m}^{g}={\tilde{x}}^{r}-{\tilde{x}}^{g}$$

(24) $$y_{m}^{r}-y_{m}^{g}={\tilde{y}}^{r}-{\tilde{y}}^{g}$$

where $x_{m}^{r}$ and $y_{m}^{r}$ denote the transformed measurement, $x_{m}^{g}$ and $y_{m}^{g}$ denote the GPS measurement.

Based on the statistic properties of the systematic error, the expectations of Equations (23) and (24) are described as

(25) $$x_{m}^{r}-x_{m}^{g}=E\left[E\left[\tilde{x}\right]|r_{m},\theta_{m}\right]$$

(26) $$y_{m}^{r}-y_{m}^{g}=E\left[E\left[\tilde{y}\right]|r_{m},\theta_{m}\right]$$

To estimate the biases, a weighted nonlinear least square method is utilized in which the calculation formula is written as

(27) $$\left|\right|e^{2}\left(x\right)\left|\right|=\frac{1}{2}e^{T}We=\frac{1}{2}\sum_{i=1}^{n}w_{i}{\left[y_{i}-f_{i}\left(x\right)\right]}^{2}$$

where

$$y=\left[\begin{array}{c} x_{m}^{r}-x_{m}^{g}\\ y_{m}^{r}-y_{m}^{g} \end{array}\right],f=\left[\begin{array}{c} E[E\left[\tilde{x}\right]|r_{m}\theta_{m}]\\ E[E\left[\tilde{y}\right]|r_{m},\theta_{m}] \end{array}\right]$$

and i=1,...,n is the index of the measurement, W is the weight matrix and calculated by the inverse of the deviation.

To calculate the bias $x=(r_{b},\theta_{b})$, the gradient $▿\left|\right|e^{2}\left|\right|$ is utilized by

(28) $$\frac{\partial ||e^{2}\left(x\right)\left|\right|}{\partial x}=-\sum_{i=1}^{n}w_{i}\left[y_{i}-f_{i}\left(x\right)\right]\frac{\partial f_{i}\left(x\right)}{\partial x}$$

Hence the bias is estimated with an initial value $x_{0}$ by taking in the direction which $\left|\right|e^{2}\left|\right|$ drops most rapidly. More details of the nonlinear least square method could be found in [16].

## 4. Simulation

**Figure 1:**
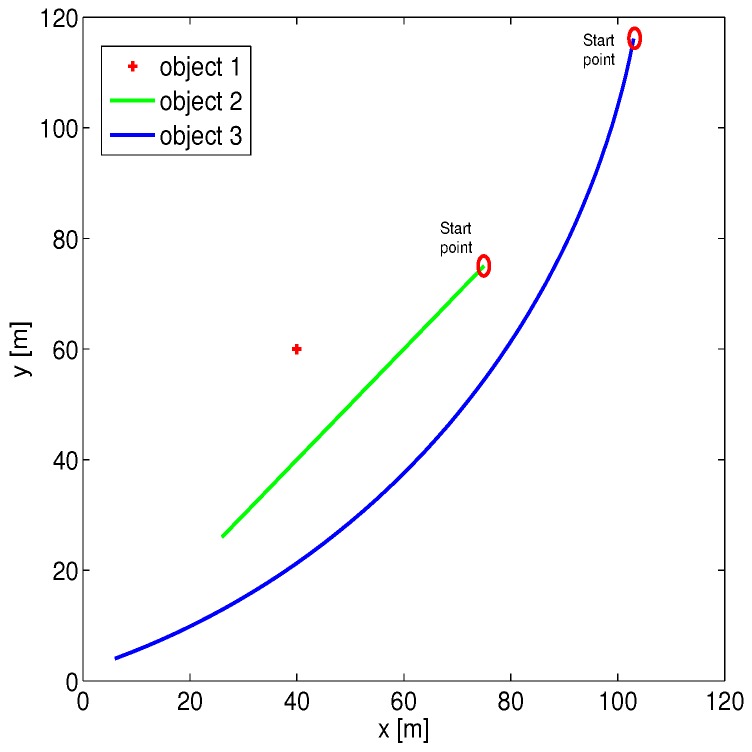
The trajectories of three objects.

In this section, simulated data were used to evaluate the effectiveness of the proposed approach. The statistic properties of the systematic error are first evaluated by conducting 1000 times Monte-Carlo simulation. During the simulation, three objects are measured in 50 steps: (1) stationary object; (2) object with constant velocity; (3) object with maneuver (constant turn). The trajectories of all objects are exhibited in Figure 1. The radar is at the original point and the biases are given by $r_{b}$ = 15 m and $\theta_{b}$ = 0.3 rad, where the corresponding random noises are given by zero mean with deviation 1 m in range and $0.1^{\circ }$ in bearing.

**Figure 2:**
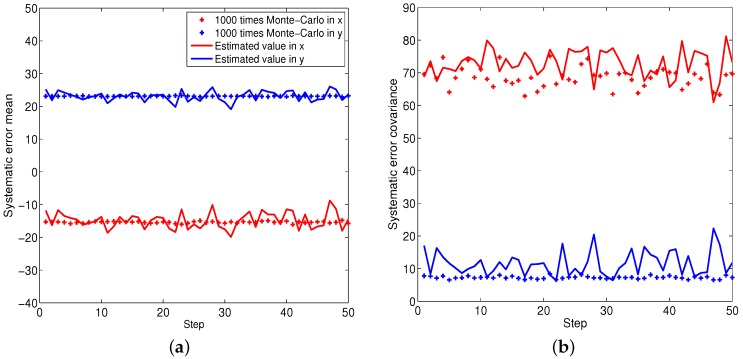
The statistic properties of the systematic error for object one. (**a**) Mean of systematic error; (**b**) Covariance of systematic error.

**Figure 3:**
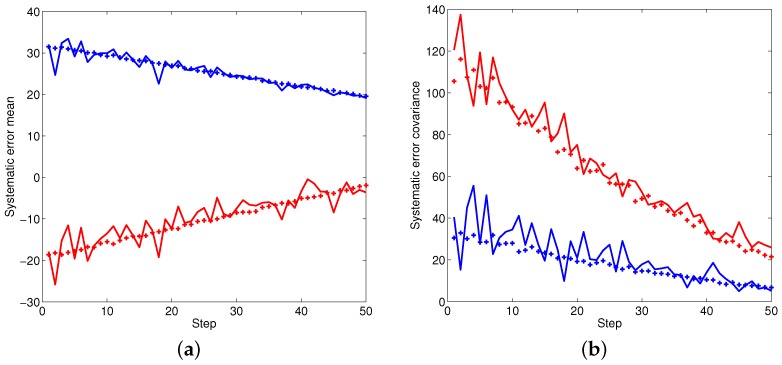
The statistic properties of the systematic error for object two. (**a**) Mean of systematic error; (**b**) Covariance of systematic error.

**Figure 4:**
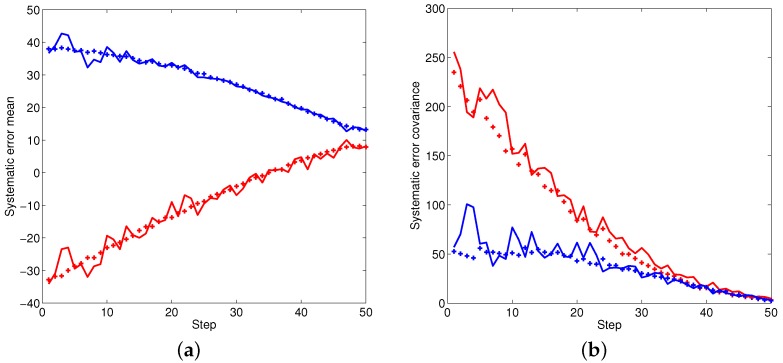
The statistic properties of the systematic error for object three. (**a**) Mean of systematic error; (**b**) Covariance of systematic error.

Figure 2, Figure 3 and Figure 4 illustrate the performance of the systematic error analyzation for all objects by using the proposed models. Since the first object is a non-moving object, both the estimated expectation and covariance of the systematic error are close to constant values. The estimation for constant velocity and maneuver objects are exhibited in Figure 3 and Figure 4. It is observed that the errors are independent with the positions. It is also observed that both the expectation and covariance are significantly decreased when objects are close to the radar.

The biases are also estimated by using the non-linear least square estimator. During the estimation phase, additional information from GPS are required (zero mean, standard deviation 10 m). Note that the sensor alignment method [6] (normal least square method, using first order Taylor extension for linearizion) is also utilized to compare the performances.

**Figure 5:**
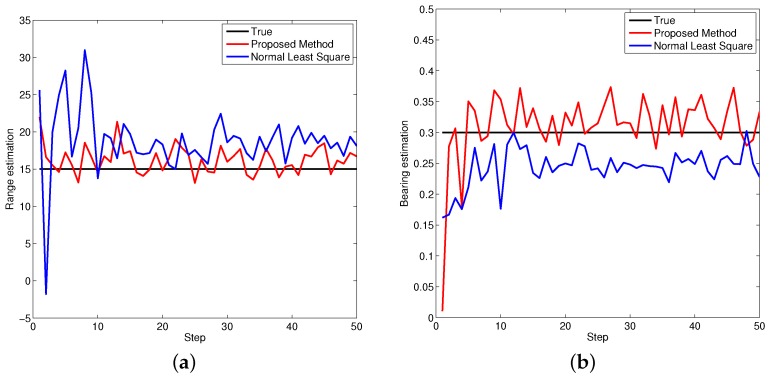
Bias estimations for object one. (**a**) Bias estimation in range; (**b**) Bias estimation in bearing.

**Figure 6:**
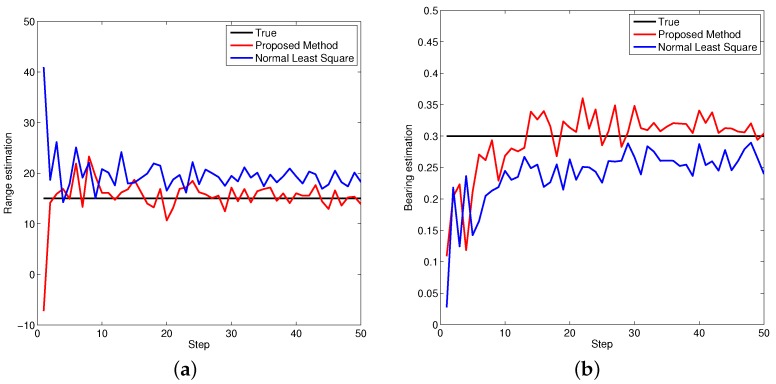
Bias estimations for object two. (**a**) Bias estimation in range; (**b**) Bias estimation in bearing.

**Figure 7:**
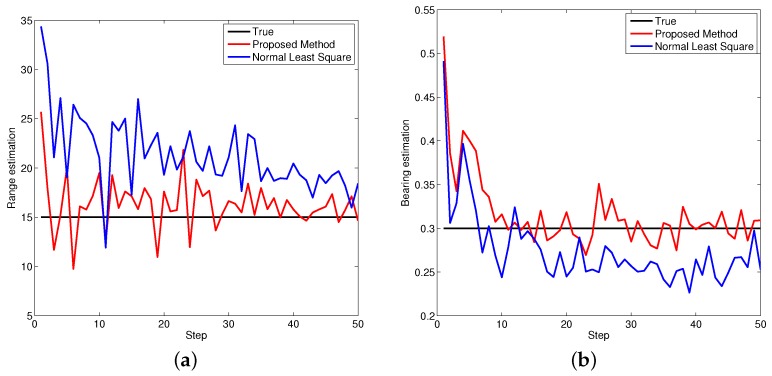
Bias estimations for object three. (**a**) Bias estimation in range; (**b**) Bias estimation in bearing.

Figure 5, Figure 6 and Figure 7 demonstrate the performance of both approaches, where the biases are recursively estimated in terms of range and bearing. Since the proposed approach operates on the raw data level, the estimation contains huge differences at beginning. With increasing time, the estimator successfully converges to the ground truths. It is observed that the proposed approach has high performance in all scenarios compared to the state-of-the-art.

**Figure 8:**
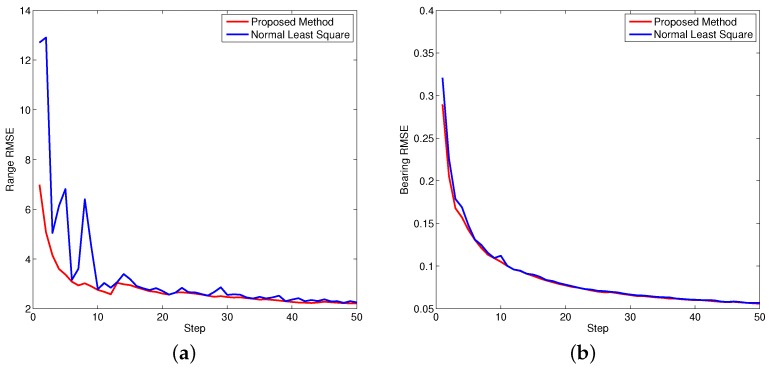
Bias root mean square equation (RMSE) for object one. (**a**) Bias RMSE in range; (**b**) Bias RMSE in bearing.

**Figure 9:**
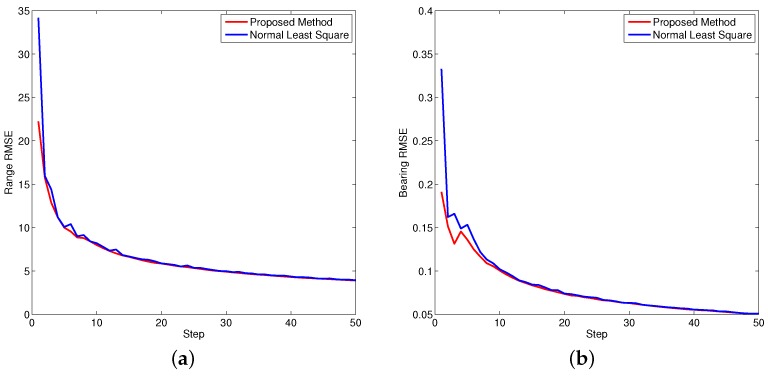
Bias RMSE for object two. (**a**) Bias RMSE in range; (**b**) Bias RMSE in bearing.

**Figure 10:**
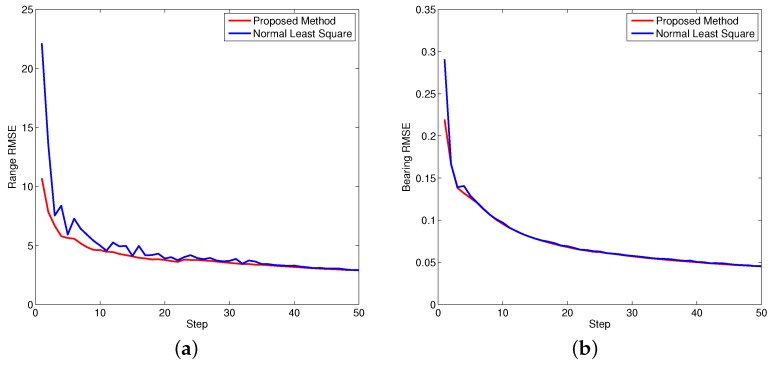
Bias RMSE for object three. (**a**) Bias RMSE in range; (**b**) Bias RMSE in bearing.

To evaluate the performance quantitatively, the root mean square equation (RMSE) is also used as follows:

(29) $$\text{RangeRMSE}=\sqrt{\frac{\sum_{i=1}^{n}{\left(\text{EstimatedRangeBias}-\text{TrueRangeBias}\right)}^{2}}{n}}$$

(30) $$\text{BearingRMSE}=\sqrt{\frac{\sum_{i=1}^{n}{\left(\text{EstimatedBearingBias}-\text{TrueBearingBias}\right)}^{2}}{n}}$$

where *n* is the step index. Figure 8, Figure 9 and Figure 10 show the performance of both approaches evaluated by RMSE. Since the bearing bias is much smaller compared to the range bias, the corresponding RMSE is smaller in contrast to the range bias. Based on the calculation, it is concluded the overall performance of the proposed approach is better than the normal least square method.

Contributions are summarized as follows: First, the statistic properties of the systematic error are analyzed and evaluated. Second, a least square method is proposed to estimate sensor biases based on the proposed statistics properties. The benefit of the proposed systematic error model is that the unbiased measurement may be calculated during the filtering phase combined with the uncertainties. Furthermore, the sensor biases could also be calculated by solely relying on the measurements.

## 5. Conclusions

In this paper, the systematic error is analyzed and modeled with respect to its statistic properties. The proposed model not only calculates the expectation of the systematic error, but also gives the covariance. Furthermore, a nonlinear least square method is proposed to estimate sensor biases. In comparison to the related work, the proposed approach recursively estimates both the error and sensor biases in absence of the ground truths. The performance is evaluated by using 1000 times Monte-Carlo simulation and three objects with different maneuvers. A comparative study has also been carried out and exhibits the high performance of the proposed approach. Future work focuses on the application of the proposed approach in real scenarios.
